# Relationship between Male Age, Accessory Gland, Sperm Transferred, and Fitness Traits in *Drosophila bipectinata*

**DOI:** 10.1673/031.013.15901

**Published:** 2013-12-25

**Authors:** H. T. Santhosh, M. S. Krishna

**Affiliations:** Drosophila stock center, Department of Studies in Zoology, University of Mysore, Manasagangotri, Mysore — 560 006. Karnataka, India

**Keywords:** fecundity, fertility, male age, copulation duration

## Abstract

The number of cells and the size of the cells in the male accessory gland, the quantity of accessory gland proteins, and their effects on fitness in males of different ages were studied in *Drosophila bipectinata* Duda (Diptera: Drosophilidae). Male age was significantly positively correlated with the size of accessory gland, the number of main cells of the accessory gland, the quantity of protein in unmated males, the duration of copulation, the transferred quantity of protein and sperm to the mated female, fecundity, and fertility, while male age was significantly negatively correlated with the size of main cell in the accessory gland and the quantity of protein in mated males. The size of the main cells was significantly positively correlated with the quantity of protein in unmated males but significantly negatively correlated with the size of the accessory gland and the number of main cells in the accessory gland. These results suggest that *D. bipectinata* young males, with their smaller size of their accessory glands and having fewer and larger main cells in their accessory glands, produced the least quantity of protein and transferred significantly less protein and sperm to the mated female than did middle and old age males. Thus, this study suggests that in *D. bipectinata*, male age affects the number of accessory gland cells and the quantity of protein in the accessory gland. The size and number of main cells in the accessory gland and the size of the accessory gland were related to the production of protein. Females who mated with old males obtained a fitness benefit.

## Introduction

In male insects, the accessory gland of the reproductive system develops from a separate set of cells in the genital imaginal disc (Nothiger et al. 1977). The secretions of this gland are transferred to the female during copulation along with sperm (Chen 1984). In the mated female accessory gland, secretion has been shown to induce physiological changes, such as stimulation of oviposition, egg laying, reduction in female receptivity to courtship, facilitation of sperm storage, and maintenance of sperm in the mated female (Herndon and Wolfner 1995; Chen 1996; Tram and Wolfner 1999; Chapman et al. 2000; Heifetz et al. 2000).

Studies of the accessory gland in *Drosophila* have provided information on its ultrastructure, chemical composition, and physiological effects in mated females in different species of *Drosophila* (Herndon and Wolfner 1995; Chen 1996; Tram and Wolfner 1999; Chapman et al. 2000; Heifetz et al. 2000; Raviram and Ramesh 2002).

Ningegowda and Ramesh (2004), who studied different strains of *D. melanogaster* and *D. nasuta*, have shown the influence of male size on the quantity of accessory gland secretion transferred to the female. They found that larger males transfer a greater quantity of accessory gland secretion than smaller males. The mating status of males and females (Friberg 2006; Sirot et al. 2011), the effect of mating on immunity (Wigby et al. 2008), and the effect of sperm competition on male reproductive fitness (Bretman et al. 2009) have also been studied in *Drosophila.*

Male age is another important trait known to influence mating success, copulation duration, and fitness traits (Brooks and Kemp 2001; Somashekar and Krishna 2011). Different models have been proposed for female preference of male age. Some of these models suggest that females prefer to mate with young or middle age males (Beck and Powell 2000; Jones et al. 2000), while others have shown that females prefer to mate with older males (Manning 1985; Kokko and Lindstrom 1996; Kokko 1998; Avent et al. 2008). It was suggested that in species in which males do not provide parental care or any direct benefits to mated females, females prefer to mate with older males. In species of the genus *Drosophila,* males do not provide parental care or nuptial gifts to the mating female, so *Drosophila* is not suitable for testing the above hypothesis. Therefore, the present study has been undertaken in *Drosophila bipectinata* Duda (Diptera: Drosophilidae), which belongs to the *bipectinata* complex of the *ananassae* subgroup of the *melanogaster* species group (Bock 1971). The evolutionary history, reproductive isolation, and remating behavior have been studied in this species (Kopp and Barmina 2005; Matsuda et at 2005; Singh and Singh 2013). Recently in *D. bipectinata*, Somashekar and Krishna (2011) found that females preferred to mate with old males over young or middle age males, and females that mated with old males had significantly greater fecundity and fertility than females that mated with middle age and young males. However, the role of the accessory gland proteins transferred to the mated female and the measure of copulation duration, fecundity, and fertility have not been studied in *D. bipecinata.* Therefore, the present study has been undertaken in *D. bipectinata* to study male-age-related changes in the transfer of sperm and accessory gland secretions with the following objectives:

1) Whether or not the quantity of accessory gland secretions differs in different male age classes. If so, what is its association with the size and number of main cells in the accessory gland, and what is the association of the size of the accessory gland and the quantity of accessory gland proteins?
2) What is the association between duration of copulation and quantity of accessory gland secretion transferred to mated females, and what is the relationship between duration of copulation and the quantity of sperm transferred to the mated female?
3) To test the hypothesis of the relationship between male age and the quantity of the accessory gland secretion/sperm transferred to a female, i.e., that old males may transfer larger quantities of sperm, therefore requiring more time for copulation, or that old males might transfer more accessory fluid in their ejaculates due to extended copulation duration.

## Materials and Methods

The experimental stock was established from progenies of 25 isofemale lines of *D. bipectinata* collected in Mysore, India (which represents the outbred population). In each generation, 20 males and 20 females per bottle were used. These flies were cultured at 22 ± 1° C and 70% RH using wheat cream agar medium with a 12:12 L:D photoperiod. Fourth generation eggs were collected from this stock using Delcour's procedure (Delcour 1969). Eggs (n = 100) were transferred into a vial containing wheat cream agar medium. When adults started emerging, virgin females and unmated males were isolated within 3 hr of their eclosion and were then aged in the same laboratory condition.

### Assignment of age classes

Before assigning age classes to males, the longevity of unmated males of this stock was studied by transferring the males separately and individually into a vial containing wheat cream agar medium once a week and maintaining them in the same laboratory condition until their death. A total of 50 replicates were made, and mean longevity (number of days a male lived from the time of its eclosion) was recorded. Longevity of *D. bipectinata* was 58 ± 3 days. Therefore, the ages to classify young, middle-age, and old males was assigned as follows: young age = 2-3 days old, middle-age = 24-25 days old, and old = 46-47 days old. Choosing insects to be aged to each classification was performed randomly, and the insects were maintained individually in the vial containing wheat cream agar medium under the same laboratory condition as above until they were used for the experiment.

### Relationship between male age, accessory gland size, number of cells (per lobe), and cell size in accessory gland

The accessory glands consist of two types of cells, main cells and secondary cells. The main cells are very numerous and binucleate and secrete the sex peptide and acessory gland proteins. The secondary cells are located in the distal end of the glands. Their function is unknown.

**Figure 1.**
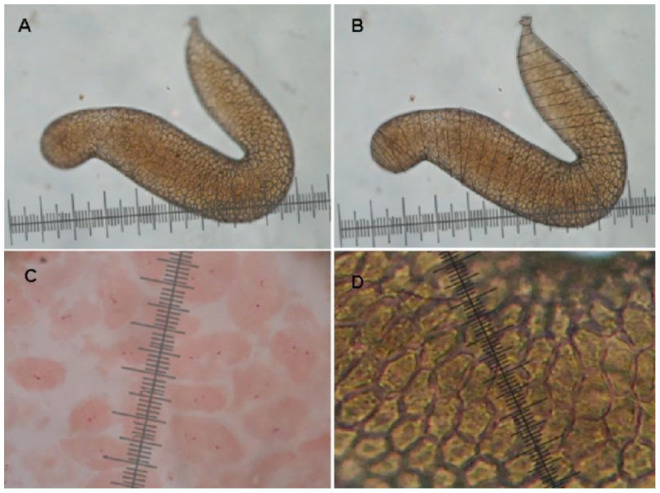
Measurement of cell number, cell size, and gland size in *Drosophila bipectinata.* A. Accessory gland lobe; B. Marked accessory gland lobe for measuring the size of the gland; C and D. Measurement of cell size of accessory gland lobe. High quality figures are available online.

The accessory glands of young, middle, and old unmated males were separately dissected out using Medium A (Ashburner 1970) and were fixed in 1N HCl for 5 min. Photographs of accessory glands were taken at 40× using a digital camera. The shape of the accessory gland in *D. bipectinata* was found to be ‘s,’ ‘c,’ or ‘j’ shaped. For the sake of convenience, the whole area of each gland was divided into smaller areas consisting of triangles, trapeziums, and rectangles (Figure 1). The areas of these geometrical forms were calculated individually (Ravi Ram and Ramesh 2002). The sum of these areas was considered as the size of the gland (in cm^2^). The actual area of the gland in the fly was calculated by dividing these values with the magnification.

Soon after taking the photographs for the measurement of accessory gland size, the accessory glands were transferred to 2% Lactoaceto orcein stain for 20 min. Before starting the experiment, the size of the accessory gland and the number and size of of the main cells were measured separately for 50 stained and unstained accessory glands. Student's *t*-test carried out on this data showed insignificant variation, suggesting that there was no effect of stain on the measurement. Then, glands were gently opened up with fine entomological needles and squashed between a slide and cover glass using 45% acetic acid so as to spread the cells in a single layer. The number of cells in each gland was counted 40× zoom using a tally counter. Soon after counting the number of main cells of an accessory gland, the same gland was used to measure the size of the main cells. Main cell length was measured from one end to another in a polygonal main cell using a micrometer at 40× zoom. A total of 50 replicates were used to calculate the accessory gland size, the number of main cells, and the size of the ac cessory gland for each age class. One-way ANOVA was carried out on the above data using SPSS 10.1 program (IBM, www.ibm.com). Pearson correlation was also carried out between male age, accessory gland size, number of main cells per lobe ,and main cell size of accessory gland.

**Figure 2.**
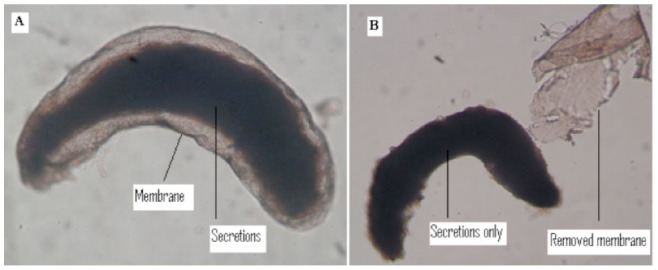
Ethanol fixed *Drosophila dipectinata* male accessory glands used for quantitative measurement of protein A. Accessory gland with the outer membrane; B. Outer membrane separated from the Acps (only secretion was used for measuring quantity of Acps). Different letters in superscript indicate significance at 0.05 levels by Tukey's post hoc test. High quality figures are available online.

### Relationship between male age and quantity of total protein in unmated males Sample preparation of unmated male.

Accessory glands of unmated (etherized) males (young, middle, and old males) were individually and separately dissected using insect saline with the help of entomological needles. These glands were fixed in 95% ethanol. Ethanol-fixed glands were taken on a clean microslide to remove the outer membrane, and only secretions of the accessory gland (Figure 2 — supporting information) were washed in a mixture of methanol and chloroform (1:1) and dried at 37° C in incubator for 15 min. About 100 μL of sample buffer (0.625 M tris-HCL pH 6.8, 1% SDS, 1% b- mercaptoethanol, and 10% glycerol) was added to each sample to dissolve the glands and secretions. Ten pairs of accessory glands from each age class were separately collected for quantitative estimation of accessory gland protein using Bradford's method (Bradford 1976).

### Quantitative estimation of protein using Bradford's method

About 50 μL of protein obtained from each of the unmated and mated males' samples as described above was separately mixed with 5 mL of Bradford reagent (100 mg CBB G-250 in 50 mL of 95% ethanol and 100 mL of 85% phosphoric acid, diluted to 1 L). The solution was allowed to stand for 5 min to develop color. The optical density of the solution was then measured using a spectrophotometer at 595 nm. The quantity of protein present in the sample was calculated by extrapolation with bovine serum albumin as the standard. The optical density of the sample was read against the blank at 595 nm. A total 50 trials were run separately for unmated male age classes (young = 50, middle = 50, old = 50).

One way ANOVA followed by Tukey's post hoc test was carried out on the data of the quantity of protein of unmated males. Pearson correlation was also carried out between male age and the quantity of protein in unmated males.

### Relationship between male age, copulation duration, quantity of protein in mated males, and transferred quantity of protein and sperm

**Sample preparation of mated males.** To obtain mated males, a five- to six-day-old virgin female and an unmated young/middle/old male were individually placed into an Elens-Wattiaux mating chamber (Elens and Wattiaux 1964) and observed for 1 hr. Pairs that remained unmated within 1 hr were discarded. If mating occured, the duration of copulation was recorded (time between initiation to termination of copulation of each pair). Soon after copulation (within 5 min), mated males were etherized and dissected to obtain Acps and fixed in 95% ethanol. The ethanol fixed glands were individually taken on a clean microslide, the outer membranes were removed as described above, and only the accessory gland secretion was taken for the estimation (Figure 2). Ten pairs of accessory glands from each age class were separately collected for quantitative estimation of accessory gland proteins using the Bradford method (Bradford 1976), as described above.

The mated females were placed individually on a glass micro slide and were dissected to remove reproductive organs including spermatheca using a stereo microscope with fine entomological needles in 20 μL of Beadle-Ephrussi saline solution (Ephrussi and Beadle 1936) (128.3 mM NaCl, 4.7 mM KCl, and 23 mM CaC12). The organs were then stained with lacto aceto orcein for 10 min, and sperm was counted using a light compound microscope at 100x.

A total of 50 trials were run separately for each of the mated male age classes for copulation duration, quantitative estimation of Acps of mated males, and transferred quantity of Acps and sperm to mated females. One-way ANOVA followed by Tukey's post hoc test was carried out on the above data. The difference in the mean value of total accessory gland proteins from unmated to mated males was considered as the transferred quantity of accessory gland proteins to the mated female. Pearson correlation was also carried out between male age, copulation duration, quantity of protein of mated males, and transferred quantity of protein and sperm to mated females.

### Relation between male age, copulation duration, transferred quantity of protein, fecundity, and fertility

To study the relationship between male age, accessory gland proteins, copulation duration, fecundity, and fertility, a 5-6-day-old virgin female and an unmated young/middle-age/old male were individually placed into an Elens-Wattiaux mating chamber and observed for 1 hr. Pairs that did not mate within 1 hr were discarded. If mating occured, the copulation duration was recorded (time between initiation of copulation to termination of copulation of each pair). Soon after copulation, mated females were individually placed into a new vial containing wheat cream agar medium every 24 hours until her death. The number of eggs laid and the number of progeny emerged were counted. The mated males were dissected in order to quantify protein, as described above.

Fifty replicates were run separately for young, middle, and old males for copulation duration, quantification of protein, fecundity, and fertility. One way ANOVA followed by Tukey's test was carried out on above data using SPSS 10.0. Pearson correlation was also carried out between the above parameters.

## Results

Table 1 shows the mean values of the number and the size of main cells in the accessory gland, the size of the accessory gland, and the quantity of protein in unmated males. The number of main cells increased with increased male age, while the size of the main cells decreased with increased male age. Whereas the size of the accessory gland and the quantity of Acps in unmated males increased from young to middle-age males, it remained the same in old males, while the size of the accessory gland decreased in old males.

One-way ANOVA followed by Tukey's post hoc test carried out on the above data showed significant variation in these traits between males of different age classes. Tukey's post hoc test showed that the mean number of main cells in the accessory gland was found to be significantly greater in old males when compared to young and middle-age males. In contrast to this, the mean size of the main cells of the accessory glands was found to be significantly greater in young males than in middle-age and old males. The size of accessory glands and quantity of protein of unmated young males was found to be significantly lower compared to middle and old males, as shown by Tukey's post hoc test. Furthermore, the size of the accessory glands was significantly greater in middle-age males than in old males, as shown by Tukey's post hoc test. The quantity of protein was insignificantly greater in unmated middle-age males than in old males.

Table 2 shows the Pearson correlation between male age, accessory gland size, the number and size of main cells in the accessory gland, and the quantity of protein in unmated males of *D. bipectinata.* Male age was significantly positively correlated with accessory gland size, the number of main cells, and the quantity of protein in unmated males, but male age was significantly negatively correlated with the size of main cells of the accessory gland. This result suggests that young males had significantly smaller accessory glands, with fewer and larger main cells in their accessory glands, and so were able to secrete less protein than middle and old males.

Table 3 shows the interaction between copulation duration and quantity of protein and sperm transferred to mated females in *D. bipectinata.* The duration of copulation and the quantity of protein and sperm transferred to the female increased with increased male age, while the quantity of protein of mated males decreased with increased age. One-way ANOVA followed by Tukey's post hoc test showed significant variation in the above traits between male age classes. Tukey's post hoc test showed that the duration of copulation and the quantity of protein and sperm transferred to mated females were significantly lower for young males than for middle and old males. Furthermore, by Tukey's post hoc test it was found that the duration of copulation and quantity of protein and sperm transferred to mated females were significantly less in middle males than in old males. The quantity of Acps in mated young males was significantly greater than in middle and old males. Similarly, Tukey's post hoc test showed that the quantity of protein was significantly greater in mated middle-age males than in old males.

**Figure 3.**
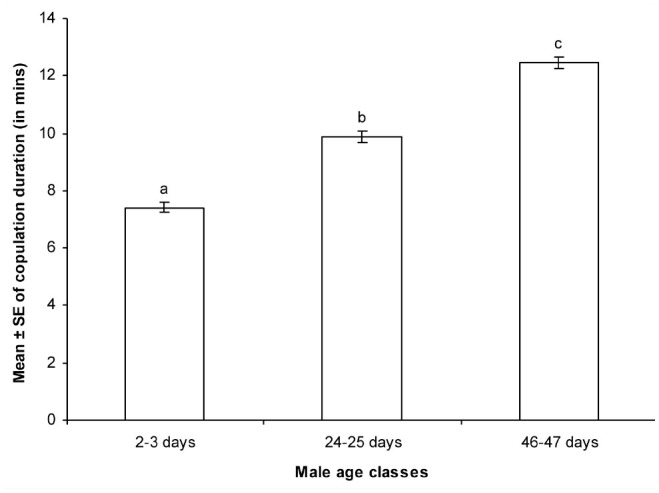
Relationship between male age and copulation duration of males of different age classes in *Drosophila bipectinata.* Different letters in superscript indicate significance at 0.05 levels by Tukey's post hoc test. High quality figures are available online.

Table 4 shows the Pearson correlation matrices of the relationship between male age and different parameters of *D. bipectinata.* It was noticed that male age was significantly positively correlated with duration of copulation and quantity of protein and sperm transferred to females. This result suggests that old males, with longer durations of copulation, transferred greater quantities of protein and sperm to the females than young and middle males did. The duration of copulation was significantly positively correlated with the quantity of transferred protein and sperm to the females.

**Figure 4.**
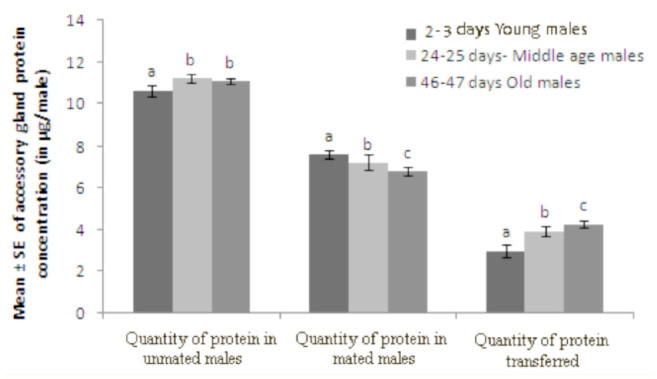
Relationship between *Drosophila bipectinata* male age and quantity of accessory gland proteins in unmated and mated insects and quantity of accessory gland proteins transferred by males of different age classes. Different letters in superscript indicate significance at 0.05 levels by Tukey's post hoc test. High quality figures are available online.

**Figure 5.**
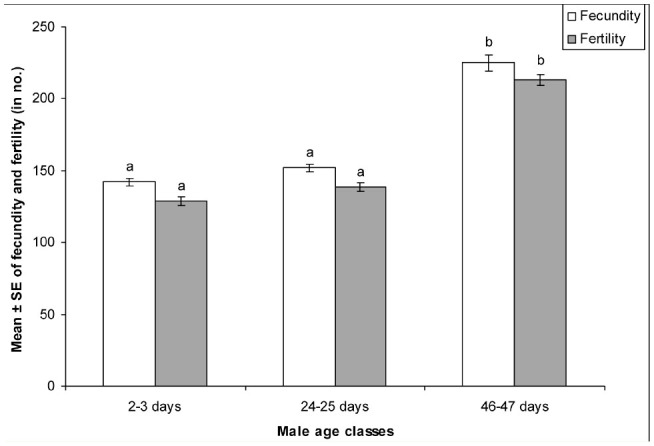
Relationship between male age and fecundity and fertility of females mated to males of different age classes in *Drosophila bipectinata.* High quality figures are available online,

Data of the relationship between male age and the duration of copulation, quantity of protein in mated males, quantity of transferred protein, fecundity, and fertility of *D. bipectinata* males are shown in Figures 3–5. It was noticed that the duration of copulation, the quantity of protein transferred to females, fecundity, and fertility increased with increased male age, while the quantity of protein of mated males decreased with increased male age. One-way ANOVA followed by Tukey's post hoc test showed significant variation in these traits between male age classes (Table 5). Tukey's post hoc test showed that young males had a significantly shorter duration of copulation and transferred a lower quantity of protein to the females. Females who mated with young males had significantly lower fecundity and fertility than those who mated with middle or old males. Furthermore, middle males had a significantly lower duration of copulation and transferred a lower quantity of protein to the females. Females who mated with young males had significantly lower fecundity and fertility than those who mated with old males, as shown by Tukey's post hoc test. The young males had a significantly greater quantity of protein when compared to middle-age and old males using Tukey's test.

Table 6 shows the correlation matrices of the relationship between different parameters of *D. bipectinata.* Male age was significantly positively correlated with duration of copulation, the transferred quantity protein, fecundity, and fertility, suggesting that young males with shorter durations of copulation transferred a lower quantity of protein and produced significantly less eggs and progeny, while old males, with their longer durations of copulation, transferred a greater quantity of protein and as a result produced a greater number of progenies. Male age was significantly negatively correlated with quantity of protein in mated males. Furthermore, duration of copulation was significantly correlated with the transferred quantity of protein, fecundity, and fertility.

## Discussion

Male age was significantly positively correlated with quantity of protein of unmated males. The highest quantity of protein was found in middle-aged males while least quantity was found in young males; this shows that age related changes in the quantity of protein occur in unmated males of *D. bipectinata.* It is presumed that differences in the quantity of protein in young, middle-aged and old unmated males could be due to the difference in the size of the accessory gland or number / cell size of the main cells in the accessory gland or the difference in the secretory activity of the accessory gland with male age. Tables 1 and 2 show that in *D. bipectinata,* the size of the accessory gland was significantly positively correlated with male age and quantity of protein in unmated males, suggesting that middle-aged or older males with greater size of accessory glands had produced greater quantities of protein, while young males with smaller size of accessory glands had produced the least quantity of protein. Further it was also found that the size of accessory glands in old males decreased significantly when compared to that of middle-aged males, but the quantity of protein was found to be more or less the same in both middle-aged and old males. This shows that the size difference in the accessory glands did not affect the quantity of protein produced by middle-aged and old males. Further, in terms of accessory gland size, it seems that the way measurements were done in the present experiment, a full and thus distended gland lumen will increase the gland's overall apparent size. Thus, males with large amounts of accessory gland protein will have larger glands simply as a consequence of having a larger amount of stored secretion, and therefore measures of protein and gland size are not independent. Our study in *D. bipectinata* supports the work of Raviram and Ramesh in *D. nasuta* (2002). They also found a positive relationship between the size of the accessory gland and quantity of protein.

In the present study, the number and size of main cells in the accessory gland were also measured to understand their relationship to protein. Tables 1 and 2 show that in *D. bipectinata*, the number of main cells in the accessory gland was significantly positively correlated with male age and quantity of protein, while the size of the main cells was significantly negatively correlated with male age and quantity of protein. Careful observation of Tables 1 and 2 show that old males, with numerous smaller main cells in their accessory glands, produced greater quantities of protein, while young males, with fewer larger main cells in their accessory glands, produced the least protein. Thus, these studies in *D. bipectinata* suggest that the number and size of the main cells in the accessory gland and the size of the accessory gland play an important role in the production of protein. In contrast to this result, in *D. nasuta,* Ravi Ram and Ramesh (2002) found a lack of influence of size of the main cells of the accessory gland on the quantity of protein synthesis. Monsma et al (1990) revealed that in *D. melanogaster* the synthesis of two specific accessory gland proteins, namely msp355a (Acp26Aa) and msp355b (Acp26Ab), were developmentally regulated. Ravi Ram and Ramesh (2002) suggested that the quantity of protein may depend on the secretory activity of cells in the accessory gland. Thus in *D. bipectinata*, the difference in the quantity of protein in unmated males of different male age classes could be due to variations in main cell number and size with male age.

In our study, the same pair of flies involved in copulation were allowed to complete copulation, then they were used to asses the quantity of transferred protein and sperm to the female to understand the relationship between male age, copulation duration, and the amount of protein and sperm transferred (Tables 3 and 4). Old males copulated longer than young or middle-age males. The reason why old males copulated longer is unknown. There are theoretical reasons to expect that old males that have not encountered females for many days would invest more resources in the first female they encounter (Parker 1970; Wedell et al. 2002). This result could also be explained by three other hypotheses. First, old males may be unable to rapidly transfer sperm and hence require longer copulations. Second, older males may transfer larger quantities of sperm, therefore requiring more time (Table 3 and Figures 3 and 4). Third, old males might transfer more accessory fluid in their ejaculates during extended copulations. The first explanation suggests that old males are worse at transferring sperm than young males. The second and third explanations suggest that old males invest more resources per mating. Tables 3 and 4 show that male age was positively correlated with duration of copulation and the quantity of protein and sperm transferred to females, suggesting that in *D. bipectinata*, old males with longer durations of copulation transferred greater quantities of protein and sperm to the females, which would fit the second and third theoretical explanation. Young males, with their shorter durations of copulation, transferred significantly less protein and sperm to females.

The same pairs of flies used in copulation were also used to record copulation duration, fecundity, and fertility, and data of these parameters are provided in Figures 3–5 and Tables 5 and 6. Male age was significantly negatively correlated with duration of copulation, while male age had a significant positive correlation with the quantity of protein transferred to the females, fecundity, and fertility, suggesting that male age has significant influence on all these parameters. Tables 3–6 and Figures 3–5 show that in *D. bipectinata*, old males with longer copulation duration transferred a greater quantity of protein, and as a result, females who mated with older males had significantly greater fecundity than females who mated with middle-age or young males. Our study supports the idea of the role of accessory gland proteins in egg production (Wolfner 1997). The greater the quantity of protein transferred to the females, the greater the egg production. The results also confirm the role of accessory gland secretion in post-mating physiological changes in the females, i.e., receptivity of females, fecundity, and fertility (Chen et al. 1996; Wolfner 1997). In the present study on *D. bipectinata*, females who mated with old males received more protein and more sperm, allowing them to have a higher fecundity and produce more progeny than females who mated with young and middle-age males (Tables 3 and 4, Figure 5). This result could be why females of *D. bipectinata* prefer older males more than middle-age and young males, as was noticed in an earlier study on three different geographical strains (Prathibha and Krishna, 2011; Somashekar and Krishna 2011). Although only one strain was used in our present study, because the female preference for old males was found in all the three geographical populations of *D. bipectinata* in other studies, the results of the present study in relation to accessory gland variation in young, middle, and old males and its effect on fecundity and fertility could also be extended to other strains. Thus, these studies suggest the occurrence of male-age-related variation in structure and quantity of protein in the accessory gland in *D. bipectinata.* The size and number of main cells in the accessory gland and the size of the accessory gland were important for production of protein. Females who mated with old males obtained fitness benefits.

**Table 1.**
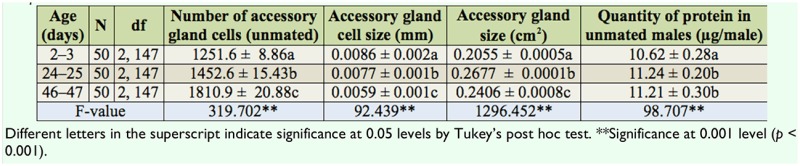
Relationship between male age and the accessory gland size, number of main cells, and size of main cells in *Drosophila bipectinata.*

**Table 2.**
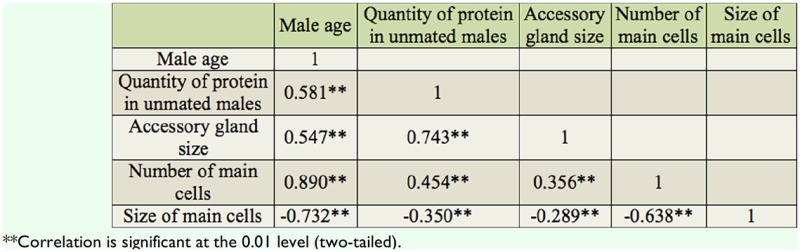
Pearson correlation between different parameters of *Drosophila bipectinata.*

**Table 3.**
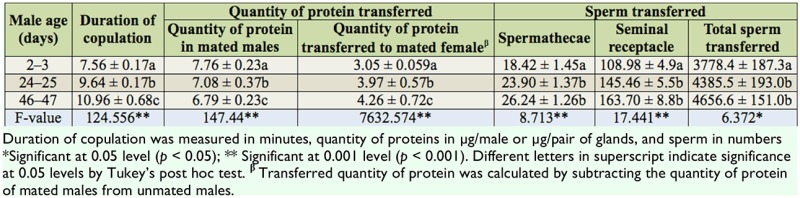
Male age relationship with different parameters of *Drosophila bipectinata.*

**Table 4.**
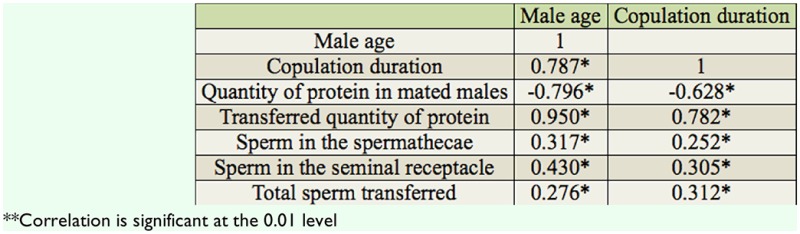
Pearson correlation between male age relationship with different parameters of *Drosophila bipectinata.*

**Table 5.**
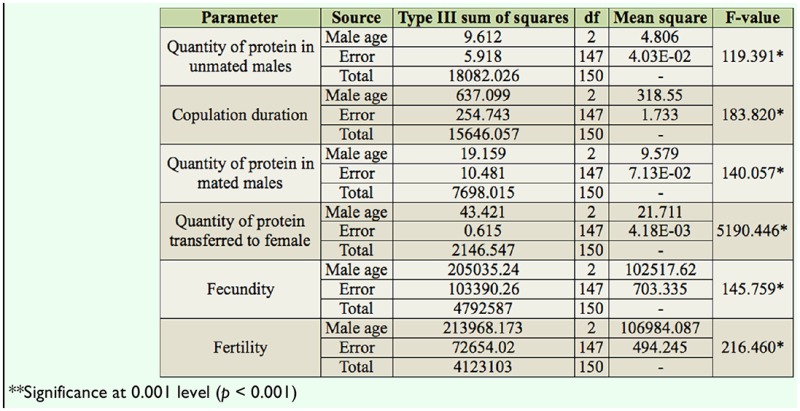
One-way ANOVA of the relationship between male age and quantity of protein in unmated and mated males, quantity of protein transferred by males, copulation duration, fecundity, and fertility of different age classes in *Drosophila bipectinata.*

**Table 6.**
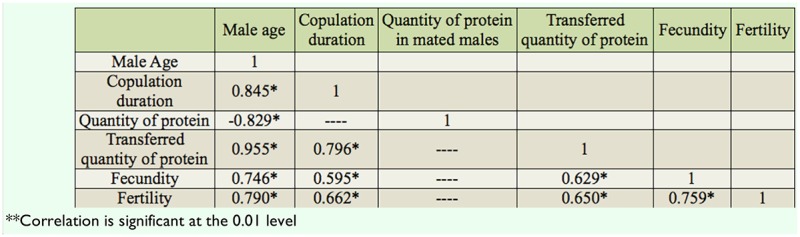
Pearson correlation between different parameters of *Drosophila bipectinata.*

## References

[bibr01] Ashburner M. (1970). Patterns of puffing activity in the salivary gland chromosomes of *Drosophila.* V. Responses to environmental treatments.. *Chromosoma*.

[bibr02] Avent TD, Price TAR, Wedell N. (2008). Age based female preference in the fruit fly *D. pseudoobscur a.*. *Animal Behaviour*.

[bibr03] Beck CW, Powell LA. (2000). Evolution of female mate choice based on male age: are older males better mates?. *Evolutionary Ecological Research*.

[bibr04] Bock IR. (1971). Taxonomy of the *Drosophila bipectinata* species complex.. *University of Texas Publications*.

[bibr05] Bradford MM. (1976). A rapid and sensitive method for the quantitation of microgram quantities of protein utilizing the principle of protein-dye binding.. *Analytical Biology*.

[bibr06] Bretman A, Fricke C, Chapman T. (2009). Plastic responses of male *Drosophila melanogaster* to the level of sperm competition increase male reproductive fitness.. *Proceedings of the Royal Society B: Biological Sciences*.

[bibr07] Brooks R, Kemp DJ. (2001). Can older males deliver the good genes?. *Trends in Ecology and Evolution*.

[bibr08] Chapman T, Neubaum DM, Wolfner MF, Partridge L. (2000). The role of male accessory gland protein Acp36DE in sperm competition in *Drosophila melanogaster*.. *Proceedings of the Royal Soceity B: Biological Sciences*.

[bibr09] Chen PS. (1984). The functional morphology and biochemistry of insect male accessory glands and their secretions.. *Annual Review of Entomology*.

[bibr10] Chen PS. (1996). The accessory gland proteins in male *Drosophila:* structural, reproductive, and evolutionary aspects.. *Experientia*.

[bibr11] Delcour J. (1969). A rapid and efficient method of egg collecting.. *Drosophila Information Service*.

[bibr12] Ephrussi B, Beadle GW. (1936). A technique of transplantation for *Drosophila*.. *The American Naturalist*.

[bibr13] Elens AA, Wattiaux JM. (1964). Direct observation of sexual isolation.. *Drosophila Information Service*.

[bibr14] Friberg U. (2006). Male perception of female mating status: its effect on copulation duration, sperm defence and female fitness.. *Animal Behaviour*.

[bibr15] Heifetz Y, Lung O, Frongillo EA, Wolfner MF. (2000). The *Drosophila* seminal fluid protein Acp26Aa stimulates release of oocytes by the ovary.. *Current Biology*.

[bibr16] Herndon LA, Wolfner MF. (1995). *A Drosophila* seminal fluid protein, Acp26Aa, stimulates egg laying in females for 1 day after mating.. *Proceedings of the National Academy of Sciences*.

[bibr17] Jones TM, Balmford A, Quinnell RJ. (2000). Adaptive female choice for middle aged mates in a lekking sandfly.. *Proceedings of the Royal Society B: Biological Sciences*.

[bibr18] Kokko H, Lindstrom J. (1996). Evolution of female preference for old males.. *Proceedings of the Royal Society B: Biological Sciences*.

[bibr19] Kokko H. (1998). Good genes, old age and life-history trade-offs.. *Evolutionary Ecology*.

[bibr20] Kopp A, Barmina O. (2005). Evolutionary history of *D. bipectinata* species complex.. *Genetical research*.

[bibr21] Manning JT. (1985). Choosy females and correlates of male age.. *Journal of Theoretical Biology*.

[bibr22] Matsuda M, Tominura Y, Tobari YN. (2005). Reproductive isolation among geographical population of *D. bipectinata* Duda (Diptera, Drosophilaidae) with recognition of three subspecies.. *Genetica*.

[bibr23] Monsma SA, Harada HA, Wolfner MF. (1990). Synthesis of two *Drosophila* male accessory gland proteins and their fate after transfer to the female during mating.. *Developmental Biology*.

[bibr24] Ningegowda NL, Ramesh (2004). Male accessory glands in *Drosophila:* a study on relationship between quantity of secretory roteins and body size.. *Entomon*.

[bibr25] Nothiger R, Dubendorfer A, Epper F. (1977). Gynandromorphs reveal two separate primordia for male and female genitalia in *Drosophila melanogaster*.. *Development Genes and Evolution*.

[bibr26] Parker GA. (1970). Sperm competition and its evolutionary consequences in the insects.. *Biological Reviews*.

[bibr27] Prathibha M, Krishna MS, Jyaramu SC. (2011). Male age influence on male reproductive success in *Drosophila ananassae* (Diptera: Drosophilidae).. *Italian Journal of Zoology*.

[bibr28] Ravi Ram K, Ramesh SR. (2002). Male accessory gland secretory proteins in *nasuta* subgroup of *Drosophila*: Synthetic Activity of Acp.. *Zoological Science*.

[bibr29] Singh A, Singh BN. (2013). Studies on remating behavior in the *D. bipectinata* species complex: intra and interspecific variations.. *Behavioral Processes*.

[bibr30] Sirot LK, Wolfner MF, Wigby S. (2011). Protein-specific manipulation of ejaculate composition in response to female mating status in *Drosophila melanogaster*.. *Proceedings of the National Academy of Sciences*.

[bibr31] Somashekar K, Krishna MS. (2011). Evidence of female preference for older males in *D. bipectinata*.. *Zoological studies*.

[bibr32] Tram U, Wolfner MF. (1999). Male seminal fluid proteins are essential for sperm storage in *Drosophila melanogaster*.. *Genetics*.

[bibr33] Wedell N, Gage MJG, Parker GA. (2002). Sperm competition, male prudence and sperm limited females.. *Trends in Ecology and Evolution*.

[bibr34] Wigby S, Domanitskaya EV, Choffat Y, Kubli E, Chapman T. (2008). The effect of mating on immunity can be masked by experimental piercing in female *Drosophila melanogaster*.. *Journal of Insect Physiology*.

[bibr35] Wolfner MF. (1997). Tokens of love: functions and regulation of *Drosophila* male accessory gland products.. *Insect Biochemistry And Molecular Biology*.

